# The Hepatic Microenvironment and TRAIL-R2 Impact Outgrowth of Liver Metastases in Pancreatic Cancer after Surgical Resection

**DOI:** 10.3390/cancers11060745

**Published:** 2019-05-29

**Authors:** Lauritz Miarka, Charlotte Hauser, Ole Helm, Dörthe Holdhof, Silje Beckinger, Jan-Hendrik Egberts, Jan-Paul Gundlach, Lennart Lenk, Sascha Rahn, Wolfgang Mikulits, Anna Trauzold, Susanne Sebens

**Affiliations:** 1 Institute for Experimental Cancer Research, Christian-Albrechts-University Kiel and University Hospital Schleswig-Holstein (UKSH) Campus Kiel, Arnold-Heller-Str. 3, Building 17, 24105 Kiel, Germany; stu116132@mail.uni-kiel (L.M.); olehelm@email.uni-kiel.de (O.H.); stu118005@mail.uni-kiel.de (S.B.); sascha.rahn@email.uni-kiel.de (S.R.); atrauzold@email.uni-kiel.de (A.T.); 2 Department of General, Visceral-, Thoracic-, Transplantation- and Pediatric Surgery, Christian-Albrechts-University Kiel and University Hospital Schleswig-Holstein (UKSH) Campus Kiel, 24105 Kiel, Germany; Charlotte.Hauser@uksh.de (C.H.); Jan-Hendrik.Egberts@uksh.de (J.-H.E.); Jan-Paul.Gundlach@uksh.de (J.-P.G.); 3 Department of Pediatric Hematology and Oncology, University Medical Center Hamburg-Eppendorf, 20251 Hamburg, Germany; D.Holdhof@gmx.de; 4 Department, Research Institute Children’s Cancer Center Hamburg, 20251 Hamburg, Germany; 5 Department of Pediatrics, Christian-Albrechts-University Kiel and University Medical Center Schleswig-Holstein, Schwanenweg 20, 24105 Kiel, Germany; lennartlenk@email.uni-kiel.de; 6 Department of Medicine I, Institute of Cancer Research, Comprehensive Cancer Center, Medical University of Vienna, 1090 Vienna, Austria; wolfgang.mikulits@meduniwien.ac.at

**Keywords:** pancreatic ductal adenocarcinoma, liver microenvironment, liver metastasis, inflammation, surgery, TRAIL-R2

## Abstract

Most patients with pancreatic ductal adenocarcinoma (PDAC) undergoing curative resection relapse within months, often with liver metastases. The hepatic microenvironment determines induction and reversal of dormancy during metastasis. Both tumor growth and metastasis depend on the Tumor necrosis factor (TNF)-related apoptosis-inducing ligand-receptor 2 (TRAIL-R2). This study investigated the interplay of TRAIL-R2 and the hepatic microenvironment in liver metastases formation and the impact of surgical resection. Although TRAIL-R2-knockdown (PancTu-I shTR2) decreased local relapses and number of macroscopic liver metastases after primary tumor resection in an orthotopic PDAC model, the number of micrometastases was increased. Moreover, abdominal surgery induced liver inflammation involving activation of hepatic stellate cells (HSCs) into hepatic myofibroblasts (HMFs). In coculture with HSCs, proliferation of PancTu-I shTR2 cells was significantly lower compared to PancTu-I shCtrl cells, an effect still observed after switching coculture from HSC to HMF, mimicking surgery-mediated liver inflammation and enhancing cell proliferation. CXCL-8/IL-8 blockade diminished HSC-mediated growth inhibition in PancTu-I shTR2 cells, while Vascular Endothelial Growth Factor (VEGF) neutralization decreased HMF-mediated proliferation. Overall, this study points to an important role of TRAIL-R2 in PDAC cells in the interplay with the hepatic microenvironment during metastasis. Resection of primary PDAC seems to induce liver inflammation, which might contribute to outgrowth of liver metastases.

## 1. Introduction

Pancreatic ductal adenocarcinoma (PDAC) is still among the most lethal cancers with metastatic spread accounting for a majority of PDAC related deaths. Because most of the patients are diagnosed with an already locally advanced or metastatic stage of the disease, only 15% are suitable for surgical intervention, the only curative therapeutic option [1]. Even those patients undergoing R0 resection mostly relapse with local recurrences or hepatic metastases within months after surgery [2]. Thus, an improved understanding of metastatic outgrowth and the underlying mechanisms is critical to improve the outcomes of these patients. 

The most common target for PDAC metastasis is the liver. Early occurrence of hepatic metastasis despite successful R0 resection of the primary tumor indicates that dissemination of PDAC cells or even pre-malignant pancreatic ductal epithelial cells might be an early event in tumor development. Accordingly, several experimental and preclinical studies, along with analysis of patient derived material, support the hypothesis of early dissemination of pancreatic epithelial and carcinoma cells from the pancreas without leading to immediate outgrowth of metastases, which requires acquisition of further epigenetic and genetic alterations or alterations of the surrounding micromilieu [3,4,5,6,7].

Survival and colonization of tumor cells in the hostile liver environment apparently depends on the composition of the liver microenvironment, such as the abundance of macrophages or neutrophils [8,9,10,11]. Furthermore, emerging evidence suggests that hepatic stellate cells (HSCs) are also critically involved in establishing and maintaining a metastatic niche [8,9,10]. HSCs are key effector cells of inflammatory processes and wound healing. Upon activation these cells transdifferentiate into hepatic myofibroblasts (HMFs), which are a major source of extracellular matrix proteins, such as Collagen I, as well as inflammatory cytokines [12,13]. We could recently show that HSCs induce and maintain a dormant phenotype of PDAC cells, while HMFs enable them to escape the growth arrest, pointing to a role of liver inflammation in reversal of a dormant phenotype and metastatic outgrowth [7]. Moreover, we could identify C-X-C Motif Chemokine Ligand 8 (CXCL-8/IL-8) and Vascular Endothelial Growth Factor (VEGF) as dormancy-inducing and -reverting factors, respectively [7]. While we could delineate aging-associated inflammation as a trigger for outgrowth of liver metastases of PDAC [7], several studies provide ample evidence that surgical interventions cause inflammatory processes, thereby promoting metastatic outgrowth [14,15,16]. In line with these and our findings, a recent study by Yang et al. demonstrated that postoperatively elevated VEGF serum levels correlate with a poor cancer-specific survival of PDAC patients, while elevated levels of CXCL-8/IL-8 are associated with a favorable prognosis, indicating that surgical resection of primary PDAC modulates the profile of inflammatory mediators, thereby impacting disease progression [17].

Tumor necrosis factor (TNF)-related apoptosis-inducing ligand (TRAIL) belongs to the TNF superfamily and has governed particular interest for its ability to preferentially induce apoptosis in cancer cells while sparing surrounding non-transformed cells [18,19]. TRAIL uses its death-domain (DD) containing receptors TRAIL-R1/TRAIL-R2 to transmit apoptotic signals [20,21]. However, we and others demonstrated that these receptors are also capable of inducing non-apoptotic signal transduction pathways, such as Nuclear Factor-κB (NF-κB), mitogen-activated kinases (MAPKs), Sarcoma kinase (Src), and AKT, leading to migration, proliferation, and invasion in vitro, as well as tumor growth and metastasis in vivo [22,23,24,25,26,27,28,29,30,31,32,33]. While the latter pro-tumoral functions are thought to be mediated by cell intrinsic mechanisms, Hartwig et al. recently showed that the TRAIL-R system also promotes tumor growth by paracrine modulation of the cancer microenvironment [34]. Genetic ablation of TRAIL-R in murine PDAC cells in an endogenous PDAC mouse model reduced tumor growth and diminished metastasis, which was associated with prolonged survival [24]. Furthermore, our previous studies uncovered a metastasis-promoting role of TRAIL-R2 in pancreatic and breast cancer [24,35]. 

Since TRAIL-R2, tumor microenvironment, and surgery can all promote cancer metastasis, in the present study we aimed to understand the impact of the interplay of TRAIL-R2 and the hepatic microenvironment on the formation of liver metastases in PDAC and to determine whether this is influenced by surgical pancreatic intervention. The findings of this study provide novel insights into our understanding of the mechanisms underlying liver metastasis early after PDAC surgery.

## 2. Results

### 2.1. Knockdown of TRAIL-R2 in PancTu-I Cells Leads to Reduced Macroscopic Liver Metastases but Higher Numbers of Small Metastatic Lesions after Primary Tumor Resection in Vivo

**Figure 1:**
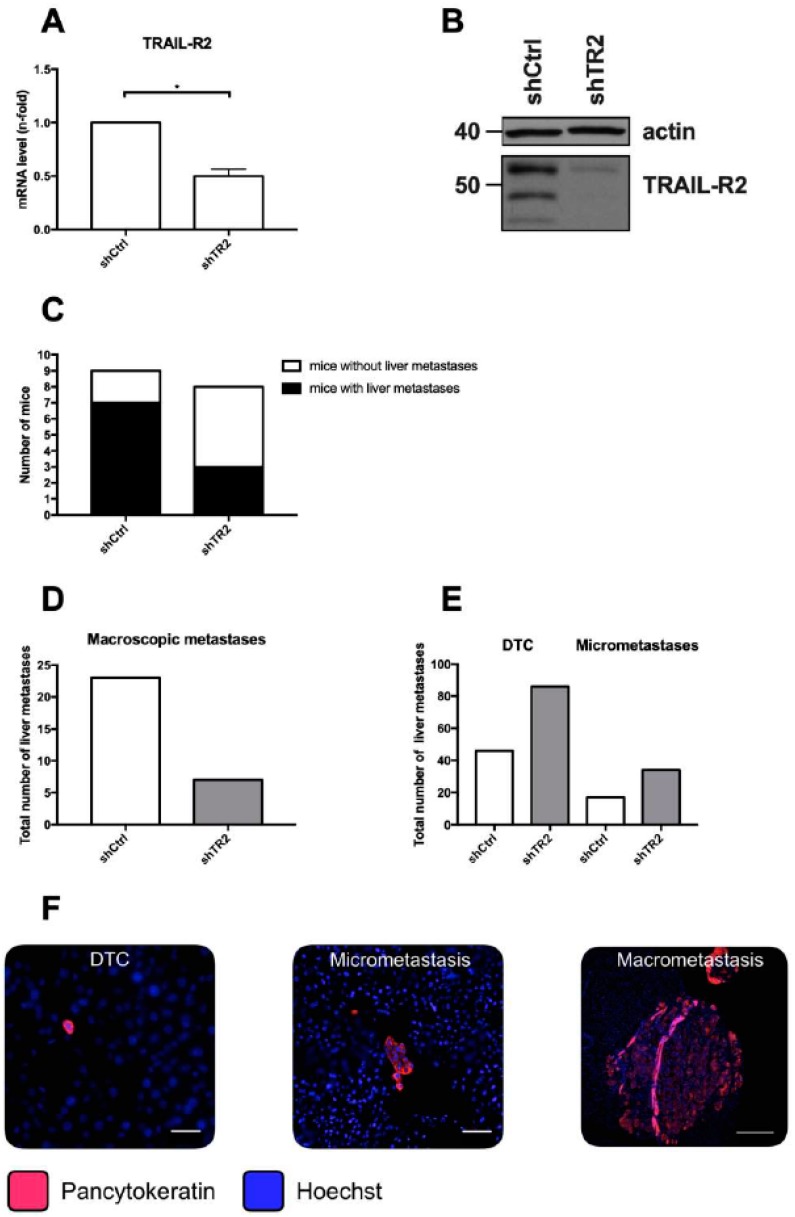
Knockdown of TRAIL-R2 in PancTu-I cells leads to reduced macroscopic liver metastases but higher numbers of small metastatic lesions after primary tumor resection in vivo. (**A**) ShRNA-mediated knockdown of TRAIL-R2 in PancTu-1 cells was verified on mRNA and protein level by (**A**) qRT-PCR or (**B**) Western blot. (**A**) The mRNA level of TRAIL-R2 was normalized to GAPDH mRNA level in control transfected (shCtrl) or TRAIL-R2 knockdown (shTR2) PancTu-I cells. Data present n-fold mRNA level of shCtrl PancTu-I cells. (**B**) A representative western blot. Actin levels were used as loading control. PancTu-I shCtrl or PancTu-I shTR2 cells were orthotopically inoculated in Severe Combined ImmunoDeficiency (SCID) beige mice and after 10 days primary tumors were resected by subtotal pancreatectomy. At 26 days post-surgery, metastatic burden in livers was assessed (**C**,**D**) macroscopically and (**E**) by immunofluorescence pan-cytokeratin staining of 3 tissue slides per animal. Disseminated tumor cells (DTC) and micrometastases were determined by counting Hoechst stained nuclei of pan-cytokeratin+ tumor cells. (**F**) Representative images of disseminated tumor cells (DTC; 1–10 cells), micrometastasis (10–100 cells), and macrometastasis (> 100 cells) are shown. Scale bars 25 µm (left), 50 µm (middle), 200 µm (right). Data represent mean ± SEM or absolute numbers of 9 (PancTu-I shCtrl) or 8 (PancTu-I shTR2) animals/group.

**Figure 2:**
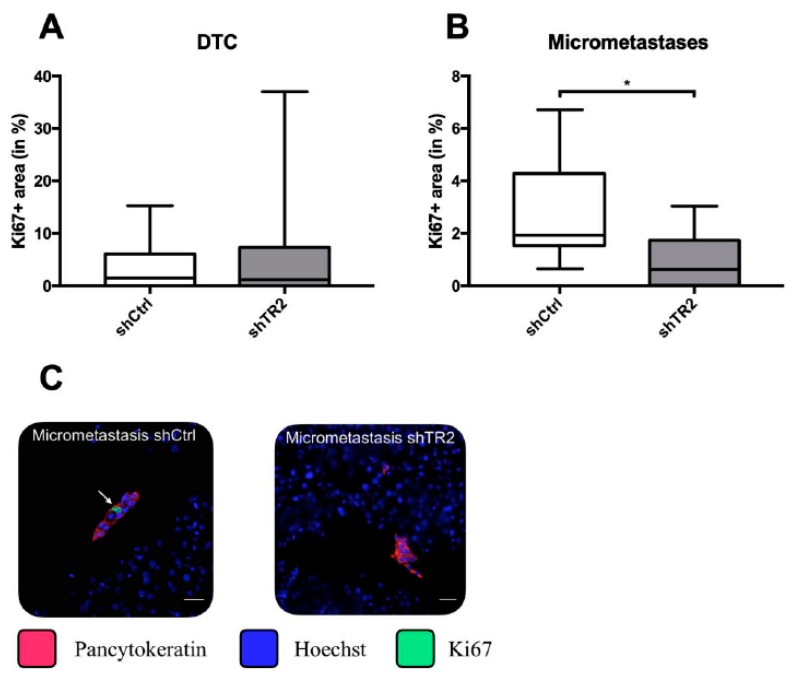
Micrometastases of animals inoculated with PancTu-I shTR2 cells show lower proportion of Ki67+ tumor cells than micrometastases of animals inoculated with PancTu-I shCtrl cells. Proportions of Ki67+ PancTu-I shCtrl and PancTu-I shTR2 cells within (**A**) DTCs and (**B**) micrometastases were analyzed by immunofluorescence staining of Ki67. (**C**) Representative images of Ki67 staining of PancTu-I shCtrl and PancTu-I shTR2 liver micrometastases. Scale bars: 25 µm. Data represent the mean ± SEM or median values with quartiles (Q_0.75_ as upper, Q_0.25_ as lower deviation) of 9 animals/group; * = *p* < 0.05.

Previous studies revealed that overall or nuclear TRAIL-R2 expression correlates with lymph vessel invasion, as well as a reduced overall survival after tumor resection in PDAC patients [24,36]. Furthermore, inhibition of TRAIL-R2 expression markedly reduces primary tumor formation in an orthotopic PDAC xenograft model, as well as the number of macroscopic liver metastases following primary tumor resection [24,36]. To elucidate the impact of the liver microenvironment on outgrowth of liver metastases in dependence on TRAIL-R2, we used our well-established clinically adapted PDAC resection model [24,37,38,39] and the PDAC cell line PancTu-I, in which TRAIL-R2 expression was stably reduced (PancTu-I shTR2). Knockdown of TRAIL-R2 was verified on mRNA level by qRT-PCR analysis (Figure 1A), as well as on protein level by western blot (Figure 1B), which both showed a substantial decrease in TRAIL-R2 mRNA and protein expression in PancTu-I shTR2 cells compared to control transfected cells (PancTu-I shCtrl). Ten days after orthotopic inoculation of PancTu-I shCtrl or PancTu-I shTR2 cells, primary tumors were resected by subtotal pancreatectomy, and 26-days-later mice were sacrificed. As previously shown [24], TRAIL-R2 knockdown strikingly decreased formation of macroscopic liver metastases (Figure 1C,D). Since micrometastases and disseminated tumor cells (DTCs) at distant sides are regarded as source of tumor relapse after surgery [40,41], micrometastatic burden in the liver was further analyzed in more detail. Therefore, immunofluorescent staining of pancytokeratin was performed and stained tumoral lesions in the liver were categorized according to the number of pancytokeratin+ cells into DTCs (1–10 cells) and micrometastases (10–100 cells) (Figure 1E,F). In contrast to the diminished number of macrometastases, livers of mice inoculated with PancTu-I shTR2 cells exhibited higher numbers of DTCs (86 versus 46 lesions/group) and micrometastases (34 versus 17 lesions/group) than mice inoculated with PancTu-I shCtrl cells (Figure 1E). To investigate if the reduced outgrowth of PancTu-I shTR2 DTCs and micrometastases is related to a diminished proliferative activity, Ki67 expression was determined. Whereas livers from animals inoculated with either PancTu-I cell variant displayed a similar proportion of Ki67+ DTCs (Figure 2A), PancTu-I shTR2 micrometastases showed a considerably less proliferative phenotype indicated by the lower proportion of Ki67+ pancytokeratin+ cells (Figure 2B,C). Altogether, these data suggest that the knockdown of TRAIL-R2 restrains progression of small metastatic lesions in the liver and suppresses tumor cell proliferation, thereby reducing outgrowth of visible macroscopic metastases after surgery.

### 2.2. Surgery Triggers a Local Inflammatory Response in the Liver in Vivo

**Figure 3:**
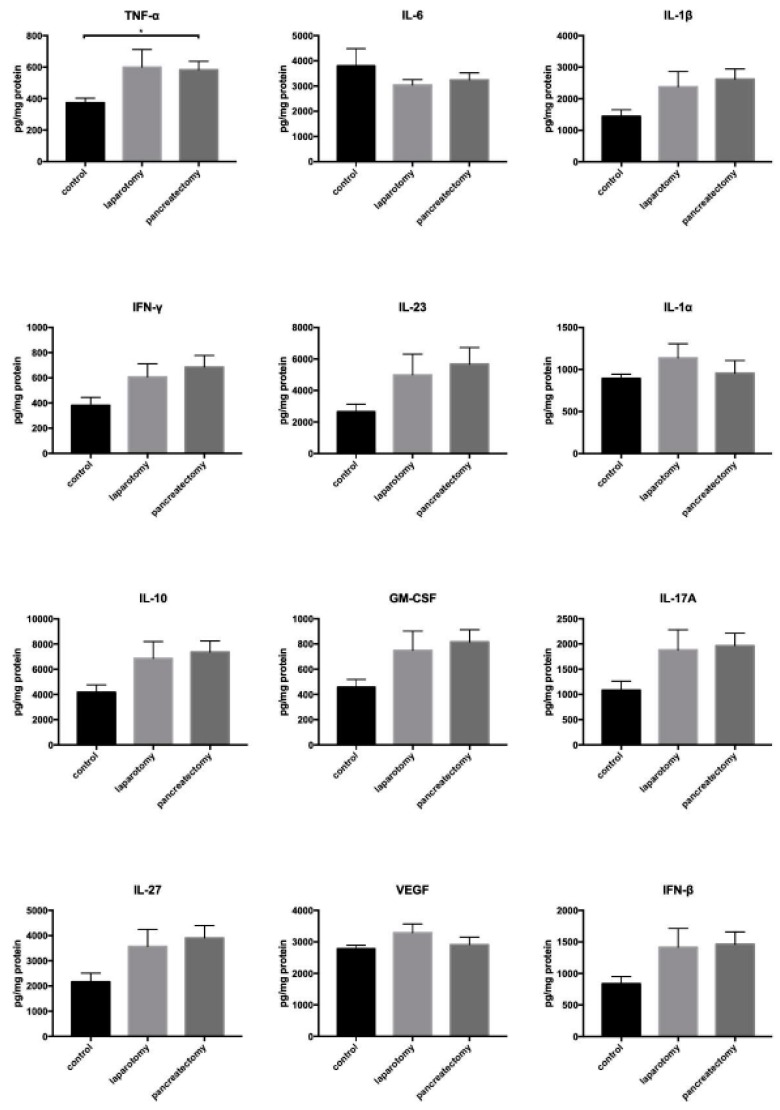
Abdominal surgery triggers a local inflammatory response in the liver. No surgery as control (*n* = 4), explorative laparotomy (*n* = 4), or subtotal pancreatectomy (*n* = 8) was performed with SCID beige mice and mice were sacrificed 48 hours after surgery to determine inflammatory cytokines in liver tissue homogenisates by LEGENDplex^TM^ multiplex analysis. Detected cytokine concentrations were normalized to protein levels of corresponding samples. Data represent the mean ± SEM of 4 or 8 animals/group; * = *p* < 0.05.

Our previous studies using the PDAC resection model showed that inhibition of systemic inflammation after primary tumor resection efficiently diminished metastatic burden in the liver [37,38], arguing for an important role of inflammation in PDAC liver metastasis. Hence, we next investigated whether abdominal surgery in general or a subtotal pancreatectomy induces not only a systemic but also a local inflammatory response in the liver. For this purpose, mice underwent an explorative laparotomy or a subtotal pancreatectomy, as performed in the resection model but without inoculation of tumor cells, or were left untreated. At 48 hours after surgery, all mice were sacrificed and liver homogenates were screened for signs of inflammation. Elevated levels of key inflammatory cytokines TNF-α, Interleukin (IL)-1β, Interferon (IFN)-γ, IL-23, IL-1α, Granulocyte-macrophage colony-stimulating factor (GM-CSF), IL-10, IFN-β, IL-17A, IL-27, and VEGF were determined in liver homogenates of mice after either surgical intervention in comparison with surgery-naive mice (Figure 3). IL-6 was the only cytokine, which was expressed at lower levels in livers of operated mice in comparison with untreated mice. Overall, these observations support the hypothesis that abdominal surgery alone or with manipulation of the pancreas induces a local inflammatory response in the liver.

### 2.3. Growth Behavior of PancTu-I Cells with Differential TRAIL-R2 Expression is not Differentially Affected by M2-Macrophages in Vitro

**Figure 4:**
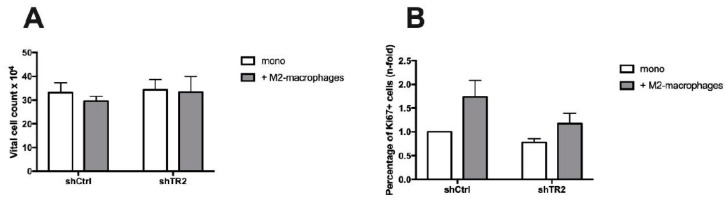
In vitro coculture with M2-macrophages does not differentially affect growth of PancTu-I shCtrl and shTR2 cells. PancTu-I shCtrl or PancTu-I shTR2 cells were indirectly cocultured in absence (mono) or presence of M2-macrophages (+ M2-macrophages) for one week. After coculture, (**A**) vital cell numbers and (**B**) Ki67 status were analyzed. Vital cell numbers were obtained by counting living cells in a Neubauer counting chamber. Ki67 status was determined by immunocytochemical Ki67 staining. Proportion of Ki67+ cells was normalized to monocultured PancTu-I shCtrl cells. Data represent the mean ± SEM of 4–5 independent experiments.

An acute liver inflammation is often accompanied by recruitment or activation of cells of innate immunity, e.g., liver resident macrophages, also termed Kupffer cells [12,42]. Emerging evidence suggests that the metastatic cascade critically depends on macrophages and that these cells can either foster or restrain outgrowth of liver metastasis [8,9,43,44]. To investigate whether macrophages are involved in the diminished outgrowth of micrometastases in mice inoculated with PancTu-I shTR2 cells as seen above, PancTu-I shCtrl cells and PancTu-I shTR2 cells were cultured for 6 days in the presence or absence of M2-macrophages, a phenotype which mostly resembles that of Kupffer cells [43,45]. Neither the presence of M2-macrophages nor modulation of TRAIL-R2 expression nor the combination of both showed an impact on the number of vital tumor cells (Figure 4A). In contrast, both cocultured PancTu-I cell variants exhibited an increased proportion of Ki67+ cells in comparison to the respective monocultured cells, indicating a higher proliferative activity of tumor cells in the presence of macrophages. However, since the difference between both cell lines was not statistically significant (Figure 4B), the interplay of tumor cells with macrophages was not further regarded in our investigations.

### 2.4. Cell Growth of PancTu-I Cells with Diminished TRAIL-R2 Expression is Reduced in the Presence of HSC in Vitro

**Figure 5:**
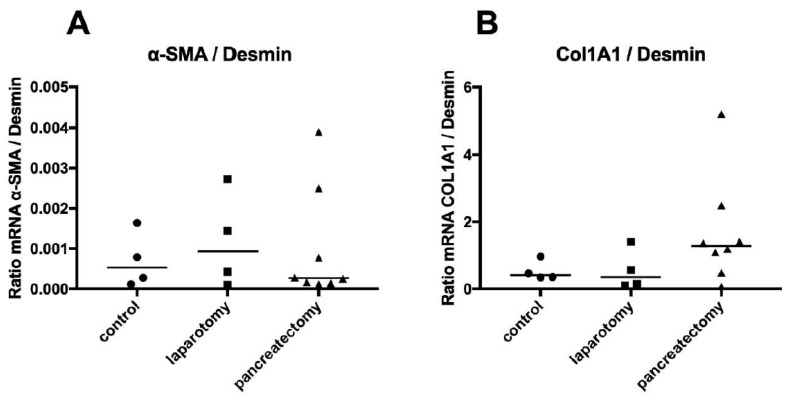
Surgery-mediated liver inflammation is accompanied by enhanced activation of HSCs into HMFs. No surgery as control (*n* = 4), explorative laparotomy (*n* = 4), or subtotal pancreatectomy (*n* = 8) was performed with SCID beige mice which were sacrificed 48 hours after surgery. RNA was isolated from snap-frozen liver tissues to determine mRNA levels of Desmin (marker for quiescent HSC), α-SMA, and Collagen 1A1 (Col1A1) (markers for HMF) by qRT-PCR. Gene expression of target genes was normalized to GAPDH gene expression. Afterwards, ratios of α-SMA to Desmin (**A**), as well as Col1A1 to Desmin (**B**), were calculated for each sample and plotted. Data represent the median and individual values from 4 and 8 animals, respectively.

HSCs and their inflammatory counterpart HMFs are key effector cells of inflammatory processes in the hepatic microenvironment [12,13]. We and others recently reported their determining role in hepatic metastasis of PDAC [7,8,9]. In order to evaluate if HSCs or HMFs might be involved in surgery-induced liver inflammation, we analyzed gene expression of HMF activation markers in liver tissues of mice after abdominal surgery or without any surgical intervention. In comparison to untreated control mice, liver tissues of mice with surgical intervention (laparotomy or subtotal pancreatectomy) revealed a higher expression ratio of genes associated with HMF activation, such as α-Smooth Muscle Actin (α-SMA) (Figure 5A) or Collagen-1A1 (Figure 5B) to expression of Desmin, the latter being associated with quiescent HSCs. These results suggest that the local inflammatory response of the liver is accompanied by activation of HSCs into HMFs.

**Figure 6:**
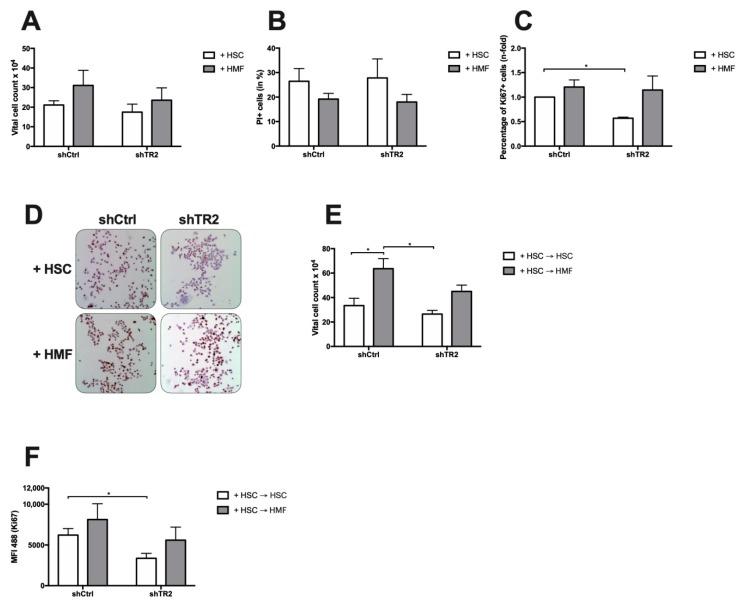
PancTu-I shTR2 cells show reduced cell growth in the presence of HSC in vitro. PancTu-I shCtrl or PancTu-I shTR2 cells were indirectly cocultured in the presence of HSC or HMF. After 6 days, (**A**) the vital cell number, (**B**) the proportion of PI+ cells, and (**C)** Ki67+ cells were determined. Vital cell numbers were obtained by counting living cells in a Neubauer counting chamber. The fraction of cells which underwent cell death was quantified by PI staining. The percentage of proliferating cells was determined by immunocytochemical Ki67 staining. (**D**) Representative images of Ki67 staining in PancTu-1 cells after different coculture conditions at 200-fold magnification. For extended coculture, HSC cocultured PancTu-I shCtrl and PancTu-I shTR2 cells were detached and re-seeded to extend the coculture either in the presence of HSC (HSC→HSC) or HMF (HSC→HMF). After another 6 days of coculture, (**E**) vital cell count and (**F**) the proportion of Ki67+ cells were assessed. The percentage of proliferating cells was determined by immunofluorescence Ki67-Alexa-488 staining. Data are expressed as median fluorescence intensity (MFI). Data represent the mean ± SEM of 3–5 independent experiments; * = *p* < 0.05.

To investigate whether HSCs and HMFs impact growth behavior of PDAC cells in dependence on TRAIL-R2 expression, PancTu-I shCtrl or PancTu-I shTR2 cells were cultured in the presence of either M1-4HSC (HSC), representing an uninflamed liver microenvironment before surgery, or M-HT (HMF), representing an inflamed hepatic microenvironment after surgery. After 6 days of coculture, the presence of HMF resulted in higher vital cell numbers of both PDAC cell populations compared to coculture with HSC. However, the number of PancTu-I shTR2 cells was lower in comparison with control PancTu-I shCtrl cells under these conditions (23.6 × 10^4^ in PancTu-I shTR2 versus 31.1 × 10^4^ in PancTu-I shCtrl), as well as after coculture with HSC (17.5 × 10^4^ in PancTu-I shTR2 versus 21.1 × 10^4^ in PancTu-I shCtrl) (Figure 6A). Propidium-Iodide (PI) staining revealed lower numbers of dead cells in the presence of HMF compared to HSC in both PancTu-I cell lines (Figure 6B), which could explain the elevated number of vital cells under inflamed conditions but did not explain the differences in vital cell numbers seen between PancTu-I shCtrl and shTR2 cells after coculture with either HSC or HMF. Importantly, PancTu-I shTR2 cells exhibited a significantly reduced proportion of Ki67+ cells after coculture with HSC compared to PancTu-1 shCtrl cells (0.53 n-fold versus 1). In contrast, the Ki67 status was similar in both cell lines after coculture with HMF (Figure 6C,D). To further support our in vivo findings, the surgery-induced inflammatory switch in the hepatic microenvironment was modeled. For this purpose, PDAC cells were first exposed for 6 days to HSC and then for another 6 days to fresh HSC or HMF. While continuous coculture with HSC (= persisting pre-surgery conditions) aggravated the non-proliferating phenotype of PancTu-I shTR2 cells, changing coculture conditions from HSC to HMF (= post-surgery conditions) reversed the HSC-mediated growth arrest in both PDAC cell lines, but resulted in considerably less vital cell numbers (Figure 6E) and a lower proportion of Ki67+ cells (Figure 6F) in PancTu-I shTR2 cells compared to PancTu-1 shCtrl cells (PancTu-I shTR2 + HSC → HMF 5591.51 vs. PancTu-I shCtrl + HSC → HMF 8120.41).

Altogether, these results suggest that HSCs (= physiological liver microenvironment) are able to impair proliferation of PDAC cells, which is further diminished by knockdown of TRAIL-R2, while HMFs (= inflamed microenvironment) promote PDAC cell proliferation, which seems to occur independently of TRAIL-R2. Furthermore, the HSC-mediated growth arrest imposed on PDAC cells with reduced TRAIL-R2 expression cannot fully be reversed by switching to HMF.

### 2.5. HSC-Mediated Growth Suppression of PancTu-I shTR2 Cells is CXCL-8/IL-8 Dependent

**Figure 7:**
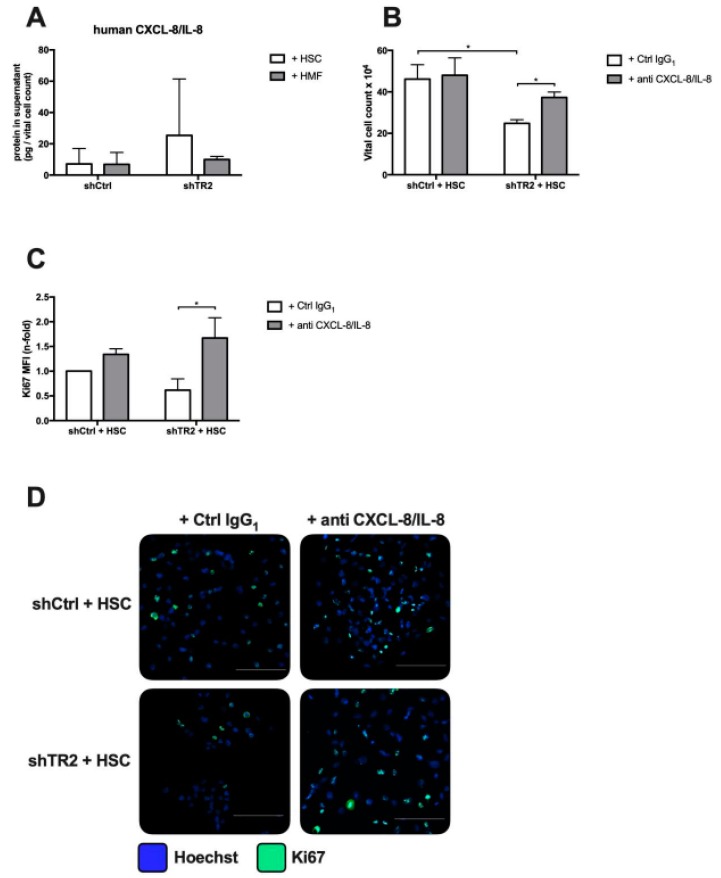
HSC-mediated growth suppression of PancTu-I shTR2 cells is CXCL-8/IL-8 dependent. Supernatants of PancTu-I shCtrl and PancTu-I shTR2 cells cocultured with HSC or HMF were analyzed for human CXCL-8/IL-8 levels by LEGENDplex multiplex analysis. (**A**) Detected CXCL-8/IL-8 concentrations were normalized to vital cell counts of corresponding PancTu-I cells. For blocking experiments, PancTu-I shCtrl and PancTu-I shTR2 cells were indirectly cocultured in the presence of HSC and treated with either 10 µg/mL control IgG1 or anti-CXCL-8/IL-8 antibody. After 6 days of coculture, (**B**) vital cell count and (**C**) the proportion of Ki67+ cells were determined. The percentage of proliferating cells was determined by immunofluorescence Ki67-Alexa-488 staining. Data are normalized to control group shCtrl + HSC + control IgG1. (**D**) Representative images of immunofluorescence Ki67 staining are shown. Scale Bars 100 µm. Data represent the mean ± SEM of 4–5 independent experiments; * = *p* < 0.05.

We next investigated how HSC impact PDAC cell growth. In order to identify soluble factors involved in this process, cell culture supernatants of HSC-cocultured PancTu-I cells were screened by LEGENDplex immunoassay. Among the secreted proteins human CXCL-8/IL-8 levels were found to be highest in supernatants from PancTu-I shTR2 cells after coculture with HSC compared to (PancTu-I shTR2 + HSC 25.34 pg/mL vs. PancTu-I shCtrl + HSC 7.16 pg/mL) (Figure 7A). CXCL-8/IL-8 has been already shown to be involved in the HSC-mediated quiescent phenotype of PDAC cells and is furthermore part of the TRAIL-induced secretome [7,34,46]. Blocking CXCL-8/IL-8 by application of an CXCL-8/IL-8 neutralizing antibody during coculture with HSC resulted in significantly higher vital cell numbers in PancTu-I shTR2 cells compared to control treated cells (PancTu-I shTR2 + anti CXCL-8/IL-8 37.33 × 10^4^ vs. PancTu-I shTR2 + Ctrl IgG1 24.85 × 10^4^; *p* = 0.0074) (Figure 7B) and increased the proportion of proliferating cells indicated by Ki67 staining intensity (Figure 7C). To confirm these findings using another PDAC cell line, coculture experiments of hepatic stellate cells with Colo357 shTR2 and Colo357 shCtrl (Figure S1A) were conducted in the presence of a CXCL-8/IL-8 neutralizing antibody or a control IgG_1_ antibody. In line with our results shown in Figure 7, blocking CXCL-8/IL-8 was able to increase the proportion of vital cells and proliferating cells in Colo357 shTR2 during coculture with HSC (Figure S1B,C).

Taken together, the data indicate that HSC-mediated growth inhibition of PancTu-I shTR2 and Colo357 shTR2 cells is dependent on CXCL-8/IL-8.

### 2.6. HMF-Mediated Proliferation Boost of PancTu-I Cells is VEGF Dependent

**Figure 8:**
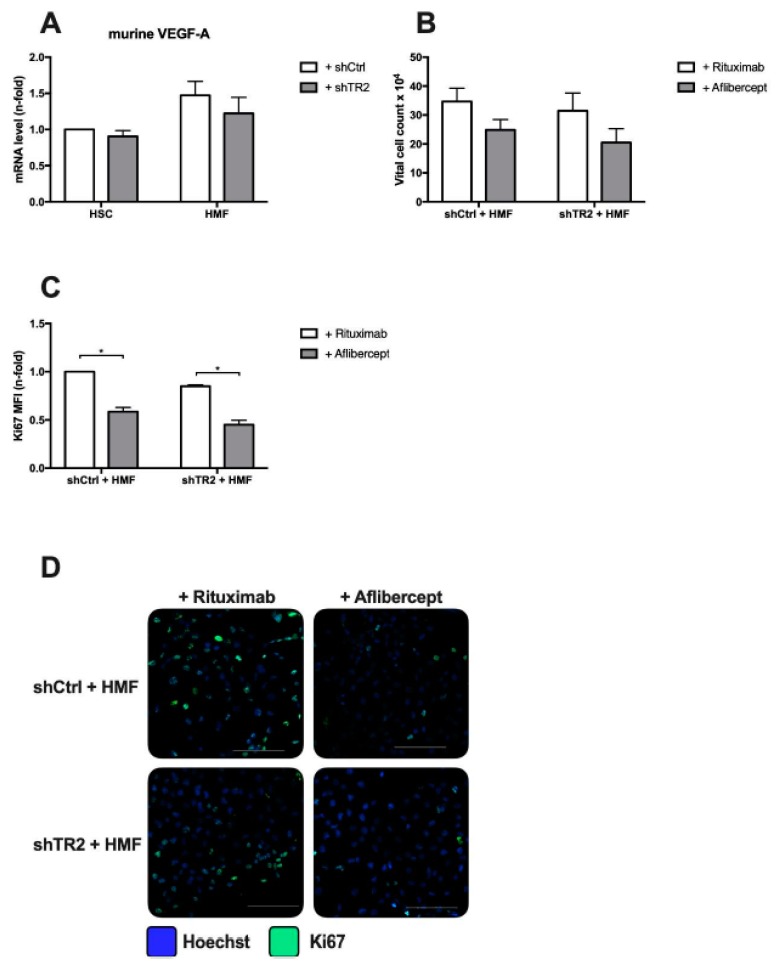
HMF-mediated proliferation boost of PancTu-I cells is VEGF dependent. (**A**) RNA was isolated from HSC or HMF after 6 days of coculture with PDAC cells and mRNA levels of murine VEGF-A were analyzed by qRT-PCR. VEGF-A gene expression was normalized to GAPDH expression and normalized expression of HSC cocultured with PancTu-I shCtrl cells was set as 1. For blocking experiments, PancTu-I shCtrl or PancTu-I shTR2 cells were indirectly cocultured in the presence of HMF and treated with 10 µg/mL of either Rituximab as control or Aflibercept. After 6 days of coculture, (**B**) vital cell count and (**C**) the proportion of Ki67+ cells were determined. The percentage of proliferating cells was determined by immunofluorescence Ki67-Alexa-488 staining. Data are normalized to PancTu-I shCtrl cells + HMF treated with Rituximab. (**D**) Representative images of immunofluorescent Ki67 staining are shown. Scale Bars: 100 µm. Data represent the mean ± SEM of 4–5 independent experiments; * = *p* < 0.05.

Next, it was investigated by which factor an inflamed hepatic microenvironment promotes proliferation of PDAC cells. Although TNF-α expression showed the strongest increase after surgical intervention (Figure 3) and inhibition of TNF-α has previously been shown to reduce liver metastasis after resection in vivo [38], blocking of TNF-α by Etanercept neither in PancTu-I shCtrl nor in PancTu-I shTR2 cells cocultured with HMF affected vital cell count or proliferation (data not shown). Since VEGF, being an HMF-released factor and shown to efficiently revert dormancy and promote proliferation of PDAC cells [7], was slightly upregulated in livers of mice after surgical intervention, gene expression of VEGF-A was determined in HSC and HMF after coculture with PancTu-I shCtrl and PancTu-I shTR2 cells. As shown in Figure 8A, gene expression of VEGF-A was higher in HMF compared to HSC but independent of the TRAIL-R2 status of PDAC cells. Accordingly, VEGF was blocked during HMF coculture by application of Aflibercept resulting in a reduced vital cell count of both PancTu-I shCtrl and PancTu-I shTR2 cells in comparison with application of Rituximab as control (Figure 8B). Furthermore, blocking of VEGF significantly diminished the proportion of Ki67+ cells of both PancTu-I cell variants by 50% (Figure 8C). 

Overall, these results again demonstrate that an inflamed hepatic microenvironment is able to promote proliferation of PDAC cells in a VEGF dependent manner. However, this effect seems to be independent of TRAIL-R2. 

## 3. Discussion

Our recent study provided ample evidence that aging-related inflammation of the liver promotes metastatic outgrowth in PDAC. This low-grade inflammation apparently does not impact homing, rather it reverts dormancy and boosts proliferation of disseminated PDAC cells in the liver [7]. These findings are in-line with other studies on PDAC demonstrating a pro-metastatic effect of inflammation in the secondary context [9,10], and further support the role of changed microenvironmental conditions by (local) inflammation of the liver as an essential driver of metastatic outgrowth. The present study extends this view by showing that abdominal surgery, which is performed to resect primary pancreatic tumors, leads not only to systemic [47,48] but also to local liver inflammation. Thus, we observed a distinct elevation of several inflammatory mediators, among them TNF-α, IL-1β, IFN-γ, GM-CSF, and VEGF, in liver homogenates of mice after surgical intervention. Particularly, for these molecules an involvement in malignancy-associated alterations, such as invasion, angiogenesis, proliferation, or apoptosis resistance, has been documented [49,50,51,52,53]. The fact that the levels of inflammatory cytokines were similar after abdominal surgery with and without pancreatectomy points to a general inflammation-promoting role of surgical interventions irrespective of organ manipulation. This assumption is supported by different studies in breast or other cancers revealing that surgery promotes early relapses and metastases formation [11,14,15]. Krall et al. showed in a murine breast cancer model that surgery leads to a systemic inflammatory response involving mobilization of myeloid cells, which promote tumor outgrowth by impairing T cell mediated tumor growth control [15]. However, they did not explicitly investigate the impact of surgery on local inflammation at the secondary site, which has been addressed in our study. The present data obtained in vivo along with coculture experiments support the view that abdominal surgery leads to (low grade) inflammation of the liver involving activation of HSCs into HMFs. While HSCs are able to restrain growth of PDAC cells, HMFs boost their proliferation, which is in line with our previous findings on other PDAC cell lines and animal models [7]. Additionally, we identified TRAIL-R2 expressed by PDAC cells as an important determinant in the tumor-stroma-interplay during hepatic metastasis of PDAC. 

We have shown previously that stable TRAIL-R2 knockdown in PDAC cells significantly reduced primary tumor growth, as well as local relapses and the number of distant metastases after resection of the primary tumor [24,36]. A detailed analysis of the liver tissue from this clinically adapted resection model revealed, however, that in contrast to the reduced number of visible macrometastases, livers of PancTu-I shTR2 inoculated animals contained more DTCs and micrometastases than livers of control animals. Importantly, particularly micrometastases were characterized by a reduced proliferative activity. These findings were substantiated by the coculture experiments demonstrating a lower number of vital and proliferating PancTu-I shTR2 cells compared to PancTu-I shCtrl cells after one week HSC coculture. However, differences between both cell lines were more apparent after HSC than after HMF coculture, indicating a more pronounced growth inhibitory effect of HSC rather than a stronger pro-proliferative effect of HMF on PancTu-I TR2 cells. Thus, to better mimic the surgery-induced inflammatory switch in the hepatic microenvironment, we first cultured both PDAC cell lines in the presence of HSC (= pre-surgery condition), and after one week, PDAC cells were either exposed to fresh HSC (= prolongation of pre-surgery condition) or HMF (= switch to post-surgery condition). This setting clearly demonstrated that proliferation of PancTu-I shTR2 cells was diminished compared to PancTu-I shCtrl cells, not only when exposed to HSC but also when conditions were switched to HMF. Overall, these data indicate that disseminated PDAC cells expressing TRAIL-R2 escape the growth control by an (uninflamed) hepatic microenvironment. Moreover, these cells show then a higher proliferative activity in a surgery-induced inflamed liver micromilieu leading to faster outgrowth of visible metastases than those PDAC cells lacking TRAIL-R2. While in our study the impact of HSCs and HMFs on growth behavior of PDAC cells in dependence on the TRAIL-R status was particularly elucidated, Hartwig et al. recently demonstrated that the TRAIL-R-induced secretome promotes the accumulation of tumor-supporting immune cells [34]. This essentially adds to other studies that describe an important role for cells of the adaptive or innate immune system in inflammation-associated maintenance or reversal of growth arrest in liver metastasis [11,54,55]. Since our experiments were performed with human PDAC cells, and therefore in immunocompromised mice, we can only speculate on the effects that are exerted by TRAIL-R2 modulated PDAC cells on immune cells of the adaptive immune system. Finally, given the tumor cell heterogeneity with respect to TRAIL-R2 expression within a tumor cell population, switching a physiological/uninflamed to an inflamed hepatic microenvironment might select for PDAC cells expressing TRAIL-R2, because these cells experience a fitness advantage under these particular conditions.

Since CXCL-8/IL-8 was markedly elevated in supernatants of PancTu-I shTR2 cells cocultured with HSC compared to cocultures with HMF or either coculture with PancTu-I shCtrl cells, CXCL-8/IL-8 was neutralized under these conditions. Accordingly, no considerable effect was observed on cell growth behavior of PancTu-I shCtrl cells, while the number of vital and proliferating PancTu-I TR2 cells was clearly elevated upon CXCL-8/IL-8 neutralization, supporting the role of CXCL-8/IL-8 as a growth restraining and dormancy mediating factor in PDAC cells [7]. These findings could be confirmed with another PDAC cell line Colo357 and are also in line with the study by Yang et al. showing that postoperatively elevated levels of CXCL-8/IL-8 correlate with a favorable prognosis and longer survival of PDAC patients after tumor resection [17]. Since our coculture system consists of murine hepatic stroma cells and human PDAC cells mimicking our in vivo xenograft model, elevated CXCL-8/IL-8 levels after coculture were of human origin, suggesting a positive feedback loop involving CXCL-8/IL-8 secretion by PDAC cells induced by stromal cells. Similar results have been obtained in our previous study, in which the human-murine coculture system was validated by a human-human coculture approach [7]. 

The mechanisms of enhanced CXCL-8/IL-8 secretion by TRAIL-R2 knockdown cells cocultured with HSC cells are still unknown. Since TRAIL-R2 knockdown cells express elevated levels of TRAIL-R1 (data not shown) and TRAIL signals in PDAC cells preferentially via TRAIL-R1 [46], it is possible that tumor cell- or HSC-derived TRAIL via binding to TRAIL-R1 is responsible for this phenomenon. Supporting the hypothesis that TRAIL-R1 induced CXCL-8/IL-8 impairs PDAC cell proliferation, recent findings implicate high TRAIL-R1 expression as a positive prognostic marker in PDAC [56], while TRAIL-R2 expression has been correlated with a worse prognosis in PDAC patients at early stages [36]. 

In accordance with the study by Yang et al., showing that postoperatively elevated VEGF serum levels are associated with poor cancer-specific survival of PDAC patients [17], and our previous study [7], we could identify VEGF as a PDAC cell proliferation promoting factor under HMF coculture (= post-surgery inflamed liver conditions). In contrast to the effects of CXCL-8/IL-8 neutralization, VEGF blockade increased the number of vital and proliferating cells in both PDAC cell lines under HMF coculture, indicating that the VEGF-mediated pro-proliferative signaling occurs in a TRAIL-R2 independent manner. This finding is further supported by the fact that the number of proliferating PancTu-I shCtrl and PancTu-I shTR2 cells were comparable after one week of HMF coculture and only differed after these conditions when both cell lines had been exposed to HSC before. 

Clinical application of neither Bevacizumab [57,58] nor Aflibercept [59] has been shown to improve overall survival of PDAC patients. However, our present data, as well as our former study [7], are not contradictory to these clinical findings because in the above mentioned studies [57,58,59] therapeutic VEGF neutralization started at advanced stages when metastases were already visible. Our studies indicate a potential benefit for such treatments at earlier stages during tumorigenesis when metastatic burden is still low and occult, aiming at prevention of metastatic outgrowth. Another strategy to prevent formation of visible and harmful metastases seems to be perioperative application of non-steroidal anti-inflammatory drugs (NSAIDs) [14,15,60]. Thus, treatment with NSAIDs along with resection of the primary tumor has been shown to diminish early relapses in breast cancer patients [14,15].

The fact that antibody mediated neutralization did not reverse the CXCL-8/IL-8 and VEGF-mediated growth arrest and its reversal, respectively, in the entire PDAC cell population suggests that both TRAIL-R dependent and independent effects on growth behavior depend on a tightly regulated balance of various pro- and anti-dormancy mediating factors. Thus, beyond CXCL-8/IL-8 and VEGF, our in vivo findings indicate a possible involvement of other inflammatory cytokines in surgery-induced liver inflammation that warrant further exploitation. 

## 4. Materials and Methods 

### 4.1. Cell Lines and Cell Culture

The human PDAC cell lines PancTu-I and Colo357 were cultured in RPMI 1640 medium supplemented with 10% FCS, 1 mM GlutaMAX, and 1 mM sodium-pyruvate (all from Life Technologies/Thermo Fisher Scientific, Waltham, MA, USA). To obtain a stable knockdown of TRAIL-R2, cells were transduced with GIPZ lentiviral shRNAmir vectors for TRAIL-R2 (PancTu-I/Colo357 shTR2) and non-silencing control (PancTu-I/Colo357 shCtrl) (GE Healthcare Dharmacon, Lafayette, CO, USA; CloneID: TRAIL-R2-shRNA: V2LHS_16711) and selected with 1 μg/mL puromycin. The murine hepatic stellate cell lines M1-4HSC and M-HT were cultured as previously described [7]. Routine cell culture and all experiments were conducted at 37°C, 5% CO_2_, and 86% humidity, and regularly checked for the absence of mycoplasma. The genotype of the cell lines was confirmed by Short Tandem Repeats Analysis. 

### 4.2. Generation of M2-Like Polarized Macrophages 

M2 macrophages were generated from monocytes of healthy donors, as previously described [61]. Informed consent was received from all donors. Monocytes were isolated from leukocyte retaining systems obtained from blood donations at the Institute for Transfusion Medicine (Kiel, Germany). First, peripheral blood mononuclear cells (PBMC) were isolated from leukocyte enriched blood donations via density gradient centrifugation. Subsequenly, monocytes were isolated from PBMC via counterflow centrifugation. Fractions with a monocyte purity of >90% were used for macrophage differentiation. For this purpose, 15 × 10^6^ monocytes were resuspended in RPMI-1640 medium supplemented with 1% FCS, 2 mM L-glutamine, 1% penicillin-streptomycin (all purchased from PAA, Pasching, Austria), and 50 ng/mL recombinant human macrophage colony-stimulating factor (M-CSF) (Bio-Legend, Fell, Germany), seeded into VueLife cell culture bags (CellGenix, Freiburg, Germany) and differentiated for 7 days at 37°C, 5% CO_2_, and 86% humidity into macrophages according to established protocols. The phenotype of anti-inflammatory M2-macrophages was validated as described previously.

### 4.3. Indirect Coculture of M2-Macrophages and PDAC Cells

For cocultures, 1 × 10^4^ PancTu-I shCtrl or PancTu-I shTR2 cells were seeded in 2 mL/well of the respective medium into a 12-well-plate. In parallel, 2.5 × 10^5^ M2-macrophages were seeded in 1 mL RPMI-1640 medium supplemented with 10% FCS, 1% L-glutamine (PAA) into a transwell insert (0.4 µm pore size, Greiner Bio-One, Frickenhausen, Germany), which was placed into another plate. After 24 hours, all media were exchanged and replaced by fresh PancTu-I medium and the transwells containing macrophages were transferred into wells with adherent PancTu-I shCtrl or PancTu-I shTR2 cells. In parallel, PancTu-I shCtrl or PancTu-I shTR2 cells were cultured alone under identical conditions (monoculture).

### 4.4. Indirect Coculture of Hepatic Stromal Cells and PDAC Cells 

One day prior to coculture, 2 × 10^4^ PancTu-I shTR2 or PancTu-I shCtrl were seeded in 6-well-plates in 2 mL medium/well, while 5 × 10^4^ M1-4HSC (HSC) or M-HT (HMF) in 1.5 mL medium/well were seeded into corresponding transwell inserts with 0.4 µm diameter pores (Greiner Bio-One). After 24 h, medium in all wells and transwells was exchanged for fresh coculture medium (RPMI 1640 supplemented with 10% FCS, 1 mM GlutaMAX, 1mM sodium-pyruvate). Afterwards, transwells were inserted into respective wells with epithelial cells. After 6 days of coculture, cells were prepared for further analysis. For extended coculture epithelial cells were counted after 6 days of coculture and 1 × 10^4^ cells were re-seeded in 6-well-plates in 2 mL fresh medium/well. Meanwhile, 5 × 10^4^ fresh M1-4HSC or M-HT in 1.5 mL medium/well were seeded into corresponding transwell inserts. After 24h, medium in all wells was exchanged for coculture medium and transwells were inserted into respective wells with epithelial cells. After 6 more days of coculture, cells were prepared for further analysis.

### 4.5. Blocking of VEGF and CXCL-8/IL-8 during Coculture

Blocking reagents were added to medium of cocultures of HSC with either PancTu-I or Colo357 cell variants, as described above, upon start of coculture and again after 72 h. Soluble CXCL-8/IL-8 was blocked using 2.5 µg/mL neutralizing human CXCL-8/IL-8 antibody (#MAB208; R&D Systems, Minneapolis, MN, USA) using monoclonal mouse IgG1 (#MAB002; R&D Systems) as isotype control. VEGF was blocked using 10 µg/mL Aflibercept (Bayer Vital, Leverkusen, Germany) using anti-CD-20 antibody Rituximab (Roche, Basel, Switzerland) as isotype control.

### 4.6. Determination of Viable Cell Number

After detachment, cells were stained with trypan blue (Sigma-Aldrich, Darmstadt, Germany) and counted using Neubauer counting chambers. For quantification of vital cells, blue stained cells were excluded from counting.

### 4.7. Propidium-Iodide Staining

For determination of dead cell, PDAC cells were stained with Propidium-Iodide (PI; Sigma-Aldrich). After 6 days of coculture, cells were detached. After centrifugation at 300 *g*, cells were resuspended in PBS supplemented with 0.5 µg/mL PI. Data acquisition was carried out on a FACSCalibur^TM^ flow cytometer (Beckton Dickinson, Heidelberg, Germany). Analysis was performed using Weasel v3.0 (The Walter and Eliza Hall Institute of Medical Research). 

### 4.8. Immunocytochemistry Staining of Ki67

Immunocytochemical staining of Ki67 to determine the proliferation status of PDAC cells after indirect coculture for 6 days was performed, as recently described [7].

### 4.9. Immunofluorescent Staining of Ki67

Immunofluorescent staining of Ki67 were carried out to investigate proliferation activity of PDAC cells after 6 days of indirect coculture. PDAC cells grown on coverslips were washed with PBS and subsequently detached. Fixation was performed by incubating coverslips with 4% Paraformaldehyde for 15 minutes at room temperature. Afterwards, cells were washed three times with PBS and incubated with ice-cold methanol for 10 minutes at −20 degrees Celsius. Coverslips were then subjected to incubation with 4% BSA/PBS for one hour at room temperature for blocking purposes. Primary antibody mouse anti-Ki67 (#556003; BD Biosciences, Heidelberg, Germany) was applied diluted in 1% BSA/PBS and incubated overnight at four degrees Celsius. After washing with PBS three times, secondary antibody Alexa Flour 488 goat anti-mouse IgG H+L (#A11029; Life Technologies) diluted 1:500 in 1% BSA/PBS was applied, as well as Hoechst 33258 (Sigma-Aldrich) diluted 1:500 for staining of nuclei. After three washings steps with PBS, slides were mounted in FluorSave Reagent (Merck Millipore, Burlington, MA, USA). Visualization was performed with a Lionheart FX automated microscope (BioTek, Bad Friedrichshall, Germany) by taking at least four random images per coverslip, using the same settings for exposure and contrast for all coverslips of an experiment. The corresponding software Gen5 was used to evaluate staining. First, thresholding of staining intensity was achieved using the control of the corresponding experiment, then staining intensity was measured by median fluorescent intensity of the staining. 

### 4.10. Detection of Cytokines in Cell Culture Supernatants

LEGENDplex^TM^ Human Inflammation Panel (Bio-Legend) was utilized to determine 13 different human cytokines/chemokines following manufacturer’s instructions. First, 2 mL of supernatants from 6 days of coculture were collected and centrifuged at 400 × *g* for 5 minutes. Cell-free supernatants were stored at −80°C until analysis. Then, 25 µL of each sample was used for analysis and analysis was carried out in duplicates. Data acquisition was carried out on a Fluorescence Activated Cell Sorting (FACS) Verse^TM^ flow cytometer (Beckton Dickinson) and analysis was done using the provided software (VigeneTech, Carlisle, MA, USA). Detected cytokine concentrations were normalized to corresponding vital cell numbers.

### 4.11. Detection of TRAIL-R2 Expression by Western Blot

Preparation of whole cell lysates, electrophoresis and Western blotting were conducted as described [36]. Primary antibodies were purchased from: ProScience Incoporated, USA (anti-TRAIL-R2 (2019)), Sigma-Aldrich (anti-β-actin (A5441)).

### 4.12. Orthotopic Xenotransplantation of Human PDAC Cells and Tumor Resection

All experiments were carried out in accordance with animal welfare and the Ministry of Energy, Agriculture, the Environment, Nature and Digitalization of Schleswig-Holstein (V312-7224.121-7(123-10/11)). Livers, resected in previously reported experiments [24] were used for analysis of DTCs and micrometastases. Briefly, 1 × 10^6^ PancTu-I shCtrl or PancTu-I shTRAIL-R2 cells were orthotopically inoculated into the pancreas of female 6-week-old SCID beige mice (CB17.Cg-Prkdc^scid^Lyst^bg-J^/Crl) purchased from Charles River (Sulzfeld, Germany). Ten days later re-laparotomy was performed and tumor-bearing pancreata were resected by subtotal pancreatectomy. Animals were sacrificed and livers were preserved in liquid nitrogen or fixed in 4.5% Phosphate Buffered Saline (PBS) buffered formalin 26 days post-resection to assess the extent of liver metastases and signs of inflammation. 

### 4.13. Laparotomy and Subtotal Pancreatectomy

Experiments were carried out in female 6-week-old SCID beige mice (CB17.Cg-Prkdc^scid^Lyst^bg-J^/Crl) purchased from Charles River. After acclimatization, laparotomy (*n* = 4) or subtotal pancreatectomy (*n* = 8) was performed, as described previously [38]. Treatment naive mice were used as control group (*n* = 4). Animals were sacrificed 48 hours after surgery and livers were preserved in liquid nitrogen. 

### 4.14. Immunofluorescent Staining of FFPE Tissue Slides

In order to determine micrometastatic burden and proliferation thereof, 3 µm slides of formalin-fixed and paraffin embedded liver specimens were deparaffinized and rehydrated in xylene and decreasing alcohol concentrations. Subsequently, the slides were subjected to antigen-retrieval in citrate buffer (pH 6.0) for 20 minutes followed by 60 minutes blocking in 4% BSA/PBS + 0.3% Triton. Afterwards primary antibody mouse anti-ki67 (#556003; BD Biosciences) was applied, diluted in 1% BSA/PBS + 0.3% Triton, and incubated overnight at 4°C. Autofluorescence was quenched by incubation in 0.1% Sudan Black B (Sigma-Aldrich) in 70% Ethanol. Following washing, slides were incubated with secondary antibody Alexa Flour 488 goat anti-mouse IgG H+L (#A11029; Life Technologies) diluted 1:500 in 1% BSA/PBS + 0.3% Triton for 1 hour at room temperature. Next, primary antibody mouse anti-pancytokeratin (#1918, Beckman Coulter, Brea, CA, USA) was applied diluted 1:100 in BSA/PBS + 0.3% Triton overnight at 4°C. For visualization, slides were subjected to incubation with secondary antibody Alexa Flour 555 donkey anti-mouse IgG (#A31570, Life Technologies) diluted 1:500 in 1% BSA/PBS + 0.3 Triton for 1 hour at room temperature. Additionally, nuclei staining was achieved by application of Hoechst 33258 (Sigma-Aldrich) and sections were mounted in FluorSave Reagent (Merck Millipore). 

### 4.15. Protein Isolation and Cytokine Detection in Murine Liver Tissues

Snap-frozen liver tissues were homogenized using the mixer mill MM 301 (Retsch, Haan, Germany) and subsequently reconstituted in PBS + 1% IGEPAL ^®^ CA-630 (Sigma Aldrich), as well as a protease inhibitor cocktail (Sigma-Aldrich) in order to avoid proteolytic degradation. After centrifugation at 10,000× *g*, supernatants were kept at −80°C until use. For determination of liver inflammation, the LEGENDplex^TM^ Murine Inflammation Panel (Bio-Legend) was utilized according to the manufacturer`s instructions. Data acquisition was carried out on a FACS Verse^TM^ flow cytometer (Beckton Dickinson) and analysis was done using the provided software (VigeneTech). Additionally, murine VEGF concentrations were determined by utilizing the Mouse VEGF Quantikine ELISA Kit (R&D Systems) according to the manufacturer’s instructions. Photometric measurements were conducted on an Infinite^R^ 200PRO Microplate Reader (Tecan, Maennedorf, Switzerland). Detected cytokine concentrations were normalized to corresponding protein values of the liver homogenates. 

### 4.16. RNA Isolation and qRT-PCR

RNA isolation and qRT-PCR was performed as previously described [7]. Primers were purchased from Realtimeprimers (via Biomol, Hamburg, Germany). Primer sequences are provided in Table 1.

### 4.17. Statistical Analysis

Sigma Plot 12.5 (Systat, Erkrath, Germany) and GraphPad Prism 7.0a (GraphPad Software, San Diego, CA, USA) were used for statistical analysis. Shapiro-Wilk test was performed for analysis of normal distribution. For parametric datasets *t*-test or repeated measures of variance analysis (one-way ANOVA RM) were executed, whereas nonparametric datasets were analyzed by Kruskal-Wallis one-way ANOVA on ranks test; *p*-Values beneath 0.05 were considered as statistically significant and indicated by an asterisk (*).

## 5. Conclusions

Importantly, surgery remains the only appropriate curative approach for PDAC patients, but the frequent outgrowth of latent micrometastatic disease in the liver after surgery still limits the patient’s outcome. This also applies to surgical interventions in borderline resectable PDAC, where vascular and multivisceral resection have to be performed [62]. In this context, strategies to increase the number of resectable PDAC cases, e.g., by neoadjuvant chemotherapy, have become a matter of debate. Based on our findings, we suggest a substantial improvement of the prognosis of PDAC patients to extend the therapeutic strategy by counteracting surgery-induced pro-tumorigenic inflammation by combining VEGF targeting antibodies or novel TRAIL-agonists and with (neo-) adjuvant chemotherapy.

## Figures and Tables

**Table 1:** Primer sequences used for qRT-PCR.

Target Gene	Sequence
Mouse α-SMA (Acta2)	F: 5’- ATG CAG AAG GAG ATC ACA GC -3’ R: 5’- CAG CTT CGT CGT ATT CCT GT -3’
Mouse Desmin	F: 5’- CAG GAG ATG GAA TAC CG -3’ R: 5’- GGC CAT CTC ATC CTT TAG GT -3’
Mouse Collagen 1A1	F: 5‘ - ATG ATG CTA ACG TGG TTC GT - 3‘ R: 5‘ - TGG TTA GGG TCG ATC CAG TA - 3’
Mouse/human GAPDH	F: 5‘ - TCC ATG ACA ACT TTG GTA TCG TGG - 3’ R: 5‘ - GAC GCC TGC TTC ACC ACC TTC T - 3‘
Mouse VEGF-A	F: 5’- ACT GGA CCC TGG CTT TAC TG -3’ R: 5’- TCT GCT CTC CTT CTG TCG TG -3’
Human TRAIL-R2	F: 5‘ - CAA TGG GGG AAG AAG AAG AA - 3’ R: 5’ - GTC CCA GCC TGT CCA TAG AT - 3’

## Supplementary Materials

The following are available online at https://www.mdpi.com/2072-6694/11/6/745/s1, Figure S1: HSC-mediated growth suppression of Colo357 shTR2 cells is CXCL-8/IL-8 dependent.

## Abbreviations

α-SMA	α-Smooth muscle actin
CXCL-8/IL-8	C-X-C Motif Chemokine Ligand 8/Interleukin-8
DTC	Disseminated tumor cell
GAPDH	Glycerinaldehyd-3-phosphat-Dehydrogenase
GM-CSF	Granulocyte-macrophage colony-stimulating factor
HMF	M-HT
HMFs	Hepatic myofibroblasts
HSC	M1-4HSC
HSCs	Hepatic stellate cells
IFN-γ	Interferon-gamma
IL-1β	Interleukin-1beta
PDAC	Pancreatic ductal adenocarcinoma
NSAIDs	Non-steroidal anti-inflammatory drugs
TNF-α	Tumor necrosis factor α
TRAIL	TNF-related apoptosis inducing ligand
TRAIL-R2	TNF-related apoptosis inducing ligand-receptor 2
VEGF	Vascular endothelial growth factor
