# Supplementary material for: The Hepatic Microenvironment and TRAIL-R2 Impact Outgrowth of Liver Metastases in Pancreatic Cancer after Surgical Resection
Source: Cancers (Basel). 2019 May 29;11(6):745. doi: 10.3390/cancers11060745 (PMC6627672; doi:10.3390/cancers11060745)
Supplement: Supplementary file 1 [file cancers-11-00745-s001.pdf]

Supplementary Materials: The Hepatic Microenvironment and TRAIL-R2 Impact Outgrowth of Liver Metastases in Pancreatic Cancer after Surgical Resection

Lauritz Miarka [1,†], Charlotte Hauser [2,†], Ole Helm [1], Dörthe Holdhof [3,4], Silje Beckinger [1], Jan-Hendrik Egberts [2], Jan-Paul Gundlach [2], Lennart Lenk [5], Sascha Rahn [1], Wolfgang Mikulits [6], Anna Trauzold [1,2,‡] and Susanne Sebens [1,‡,*]

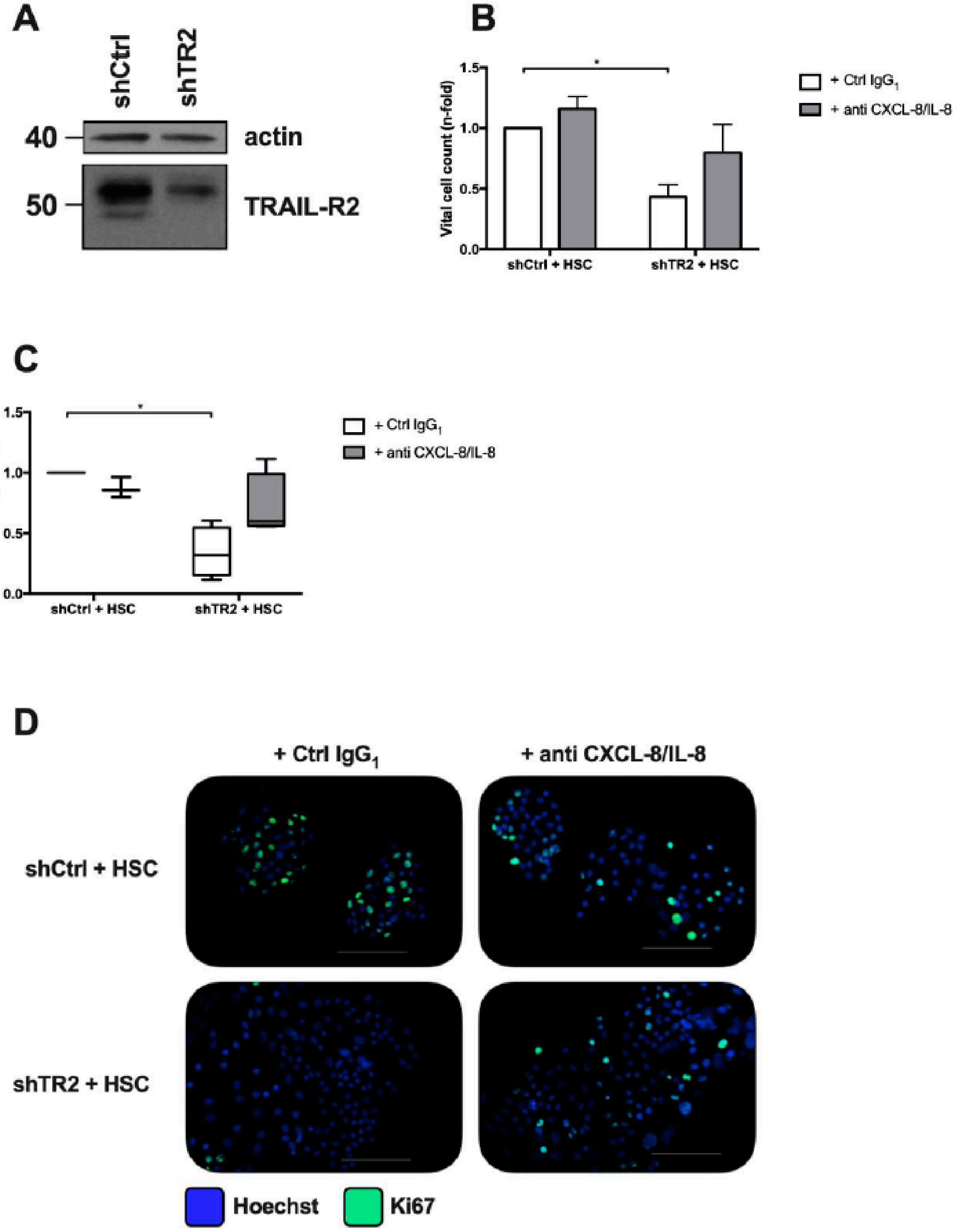

Figure S1. HSC-mediated growth suppression of Colo357 shTR2 cells is CXCL-8/IL-8 dependent. (**A**) ShRNA-mediated knockdown of TRAIL-R2 in Colo357 cells was verified on protein level by Western blot**.** Actin levels were used as loading control. For blocking experiments, Colo357 shCtrl and Colo357 shTR2 cells were indirectly cocultured in the presence of HSC and treated with either 10 µg/mL control IgG_1 or anti-CXCL-8/IL-8 antibody. After 6 days of coculture, (**B**) vital cell count and (**C**) the proportion of Ki67+ cells were determined. The percentage of proliferating cells was determined by immunofluorescence Ki67-AlexaFluor488 staining. Data are normalized to control group shCtrl + HSC + control IgG1. (**D**) Representative images of immunofluorescence Ki67 staining are shown. Scale Bars 100 µm. Data represent the mean ± SEM or median values with quartiles ($Q_{0.75}$ as upper, $Q_{0.25}$ as lower deviation) of 3–4 independent experiments; * = $p < 0.05$.
